# Trends and Factors Associated with the Non-Use of Formal Health Services in Peru, 2015–2024

**DOI:** 10.3390/ijerph23020183

**Published:** 2026-01-31

**Authors:** Miguel A. Arce-Huamani, Gustavo A. Caceres-Cuellar, Anyela Y. Guevara-Paz, Williams Carrascal-Astola, Maritza M. Ortiz-Arica, J. Smith Torres-Roman

**Affiliations:** 1 Public Health, Surveillance and Applied Research, Programa Academico de Medicina Humana, Facultad de Ciencias de la Salud, Universidad Privada Norbert Wiener, Lima 15046, Peru; gustavo2024cc@gmail.com (G.A.C.-C.); yuli94g.p@gmail.com (A.Y.G.-P.); williams.carrasca@uwiener.edu.pe (W.C.-A.); ma.ortiz.arica@gmail.com (M.M.O.-A.); 2 Escuela de Postgrado, Universidad Tecnológica del Perú, Lima 15046, Peru; jstorresroman@gmail.com

**Keywords:** good health and well-being, formal health services, epidemiology, demographic and health surveys, Peru

## Abstract

Background/Objectives: Effective use of health services is essential for universal health coverage, yet many adults in Peru still forgo formal care despite illness. Evidence describing national trends and determinants of non-use of formal health services remains limited. This study aimed to estimate national trends from 2015 to 2024 and identify factors associated with non-use among Peruvian adults. Methods: We conducted a repeated cross-sectional analysis of annual secondary microdata from the Peruvian National Household Survey (ENAHO, 2015–2024). Adults aged ≥ 18 years who reported a health problem in the last four weeks were included. Non-use was defined as not seeking care at any public or private provider (IPRESS). Survey-weighted descriptive analyses and modified Poisson regression models estimated prevalence ratios (PRs) with 95% confidence intervals, adjusting for sex, age, education, marital status, health insurance, chronic illness, disability, area, and region. Results: Among 330,165 adults, 41.5% did not use formal health services. Non-use declined until 2019, rose sharply during 2020–2021, and partially recovered thereafter. In adjusted models, non-use was lower among women (PR = 0.92; 95% CI 0.91–0.93), those with higher education (PR = 0.88; 0.86–0.90), and participants insured by EsSalud (PR = 0.65) or SIS (PR = 0.76). It was higher in the Highlands (PR = 1.07) and lower in Metropolitan Lima (PR = 0.88). Conclusions: Non-use of formal health services in Peru remains high and unequal. Expanding effective coverage, strengthening primary care, and improving health literacy are essential to achieve equitable access.

## 1. Introduction

Across Latin America and the Caribbean, the COVID-19 pandemic exposed systemic vulnerabilities in effective access, with widespread instances in which people perceived a need for care but did not obtain it [1,2]. In Chile’s universal access program (GES), 16% of beneficiaries did not use their covered benefit, largely due to trust issues, long waits, or the search for faster options, signaling persistent barriers within formal systems [3]. In Peru, health insurance expansion has improved access and narrowed socioeconomic gaps in service use, yet marked heterogeneity persists [4]. Clinically, Peruvian adults with hypertension encounter patient- and system-level obstacles that delay timely care, underscoring frictions between nominal coverage and actual utilization [5]. Globally, foregone care and reductions in preventive visits during and after pandemic surges highlight both demand- and supply-side drivers of non-use [6]. Together, these observations show that deficits in effective access endure despite formal entitlements.

Peru’s health system is structurally segmented and administratively fragmented across subsystems—MINSA/SIS, EsSalud, Armed Forces/Police services, and the private sector—each with distinct financing arrangements, provider networks, and administrative pathways. Public-sector care is mainly delivered through MINSA facilities and financially protected for poor and vulnerable groups through the Seguro Integral de Salud (SIS), while EsSalud is primarily funded by payroll contributions and provides services through its own network; smaller schemes cover the Armed Forces and Police, and private care is financed through private insurance and out-of-pocket payments [4,7,8]. Because interoperability and routine service exchange across subsystems are limited (with temporary expansions during COVID-19), “coverage” (insurance entitlement) does not automatically translate into “use” (timely contact with an IPRESS) when the reachable facility is outside the person’s scheme network or when administrative frictions and waiting times are prohibitive [5].

In addition, Peru displays marked territorial inequalities in service capacity. Hospital services are concentrated in urban areas, while primary-care facilities are more prevalent in rural regions, creating access barriers when referral capacity, staffing, and readiness are uneven across territories. These structural constraints interact with appointment systems that often rely on facility-level scheduling and generate non-trivial waiting times; for example, national evidence indicates longer delays to obtain appointments among EsSalud affiliates compared with SIS users, with variation by sex [5,9].

However, Peru-specific evidence on non-use of formal services remains scattered across constructs (e.g., service utilization, medical consultation, unmet need) [7,10] and limited time windows, which reduces comparability and makes it difficult to assess whether COVID-19–related changes persisted after the partial recovery period [11,12]. In addition, ENAHO-based studies have typically examined single years or specific subpopulations, rather than describing a decade-long trajectory using a harmonized operational definition and a consistent equity-oriented covariate set [13,14]. Consequently, it remains insufficiently characterized whether non-use of IPRESS follows stable socioeconomic and territorial gradients across 2015–2024 and which population groups contributed most to the pandemic increase and post-2022 recovery [4,12].

Conceptually, non-use of formal health services and unmet healthcare need are related but not interchangeable. Unmet need usually refers to perceiving a health need that is not satisfied, regardless of whether some form of help was sought. In contrast, in this study non-use specifically denotes not seeking care at any formal provider (IPRESS) despite reporting a recent health problem, i.e., a “no contact with the formal system” pathway. This distinction matters because the two constructs can yield different prevalence estimates and policy implications.

From a governance and health-systems perspective, persistently high non-use among adults reporting recent morbidity can be interpreted as a signal that effective coverage is failing, i.e., formal entitlements are not consistently translating into timely, acceptable, and reachable care. In this study, we define health governance as the set of institutions, rules, and decision-making processes through which health-system actors set priorities, coordinate across levels of care, allocate resources, and enforce accountability for equity and quality. Within a segmented system, governance challenges in stewardship, coordination, and execution capacity may exacerbate territorial inequities and incentivize substitution toward non-formal pathways; yet the magnitude and distribution of these dynamics in Peru over the last decade have not been quantified using nationally representative data [15,16].

What is new in our approach is the combination of a decade-long, nationally representative trend assessment (2015–2024) with an equity-oriented analysis of determinants of non-use among adults with recent morbidity.

Therefore, the objective of the present study was to estimate national trends (2015–2024) and identify factors associated with the non-use of formal health services among Peruvian adults.

## 2. Materials and Methods

### 2.1. Study Design and Setting

We conducted a repeated cross-sectional analysis using secondary microdata from the Peruvian National Household Survey (ENAHO), collected annually from 2015 to 2024 by the National Institute of Statistics and Informatics (INEI). Each ENAHO round constitutes an independent, nationally representative cross-section; therefore, all time-trend estimates reflect population-level change across calendar years rather than within-person change. ENAHO uses a two-stage, probabilistic, stratified cluster design that is nationally and subnationally representative (department, urban/rural). Primary sampling units are clusters of private dwellings in urban areas and rural census areas in rural strata; households within clusters constitute the second stage. Standardized, face-to-face interviews were carried out by trained field teams using electronic data capture. We followed the STROBE guidelines for reporting observational studies.

**Figure 1:**
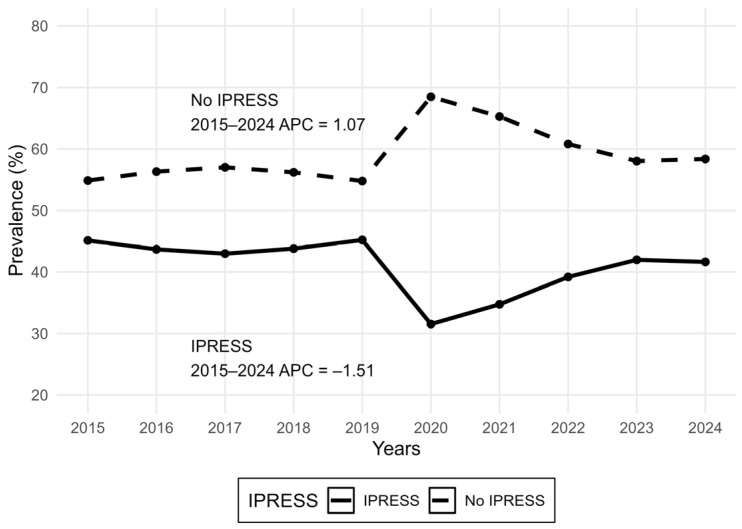
National trend in IPRESS use and non-use among Peruvian adults reporting a recent health problem, ENAHO 2015–2024. Joinpoint regression identified no statistically supported joinpoints; therefore, the Average Annual Percent Change (AAPC) equals the single-segment Annual Percent Change (APC) for 2015–2024. Overall, non-use increased by +1.07% per year, while IPRESS use decreased by −1.51% per year.

The unit of analysis was the usual resident adult (≥18 years) of sampled private dwellings. We pooled annual files from 2015 through 2024 and applied the complex survey design in all estimates so that all descriptive proportions and model-based measures reported in Table 1, Table 2 and Table 3 and Figure 1 are nationally representative.

ENAHO incorporates nonresponse adjustments in sampling weights; although design weights, stratification, and clustering were applied, differential nonresponse by geography or socioeconomic position may persist, and we acknowledge this potential bias in the Limitations.

### 2.2. Population and Sample

We included de jure adults (≥18 years) residing in sampled households who reported a health problem in the past four weeks, defined as having answered “yes” to any of the ENAHO morbidity items (p4021–p4024). Among these respondents, we classified use of formal services as having sought care at any provider establishment of the health system (Instituciones Prestadoras de Servicios de Salud, IPRESS). Specifically, “used IPRESS” was defined when any IPRESS place-of-care item (p4031–p4039) was marked (MINSA posts/centers/hospitals; EsSalud posts/polyclinics/hospitals; Armed Forces/Police facilities; private physician offices; private clinics) and was coded as nusfps = 0. “Did not use IPRESS” (non-use of formal services) was defined when none of p4031–p4039 was marked and at least one non-IPRESS option (p40310–p40313: pharmacy/drugstore, care at home, did not seek care, or other place) was marked and was coded as nusfps = 1. In ENAHO, “care at home” indicates that the respondent was managed at home rather than attending a facility; because the response does not allow us to distinguish self-care, care by household members, or a provider home visit, we classified it as non-IPRESS contact. “Pharmacy/drugstore” reflects obtaining advice and/or medicines at a retail outlet; although pharmacies are regulated, these encounters occur outside IPRESS facilities and were therefore treated as non-IPRESS contact. Thus, our outcome captures non-use of facility-based formal services (IPRESS contact) among adults reporting morbidity; it is related to but distinct from “unmet need,” which typically refers to a perceived need for care that was not satisfied regardless of the care pathway. The original Spanish wording of the ENAHO items, their English translation, and the exact recoding rules used in this study are provided in Supplementary Table S1.

Before classifying outcomes, we applied prespecified exclusions to avoid misclassification: records indicating both a pharmacy/drugstore visit (p40310 = 1) and any IPRESS visit (any of p4031–p4039 = 1) were excluded; records stating that care was not sought because it was “not necessary/not severe” (p4095 = 1) were excluded; records indicating an IPRESS place but lacking all provider information (p4041–p4047 all zero or missing) were excluded; and persons younger than 18 years were excluded by design.

Across 2015–2024, a total of 664,308 interviewed individuals were screened; after exclusions, 334,143 records were excluded, yielding a final analytic sample of 330,165 adults. For temporal description, we computed survey-weighted annual proportions of non-use and use of IPRESS across survey years (Figure 1).

### 2.3. Data Collection

Trained interviewers collected data through standardized, face-to-face surveys using electronic tablets following INEI protocols. Core modules used for this analysis covered recent morbidity and care-seeking in the last four weeks, reasons for not seeking care, type of provider and place of care, and sociodemographic characteristics. Fieldwork was conducted under centralized supervision with periodic spot checks and back-checks and with built-in electronic consistency checks in the data-capture system to ensure completeness and internal validity over 2015–2024.

### 2.4. Variables

The study outcome was non-use of formal health services (nusfps) among adults reporting a health problem in the previous four weeks. As defined in Section 2.2, respondents were classified as “used IPRESS” when they sought care at any provider establishment within the formal health system (IPRESS), and as “did not use IPRESS” when no IPRESS contact was reported and the respondent instead reported pharmacy/drugstore care, care at home (self-care and/or care by household members), no care sought, or another non-IPRESS option.

Covariates were specified a priori based on two complementary conceptual lenses: the social determinants of health and established health-services utilization models. Accordingly, we grouped covariates into predisposing characteristics (sex, age group, educational attainment, marital status, and mother tongue), enabling resources (health insurance, household poverty status, household wealth quintile, area of residence, and natural region), and need-related factors (self-reported chronic illness/discomfort and disability). Variable categories and reference groups were harmonized across 2015–2024 to ensure comparability. The original ENAHO items, their English translation, and the recoding and harmonization rules are documented in Supplementary Table S1.

### 2.5. Data Analysis

We produced survey-weighted descriptive statistics for all variables, presenting frequencies and percentages that account for the ENAHO complex sampling design. Bivariate associations between non-use of formal services and each covariate were assessed using the Rao–Scott design-adjusted chi-square test [17], and the resulting *p*-values are presented in Table 2. To estimate adjusted associations, we fitted modified Poisson regression models with a log link and robust (sandwich) standard errors to obtain prevalence ratios (PRs) and 95% confidence intervals [18]. All models incorporated sampling weights, stratification, and primary sampling units using Taylor series linearization for variance estimation. Covariates were prespecified based on conceptual frameworks (social determinants of health; health-services utilization) and retained regardless of bivariate significance to minimize residual confounding. Multicollinearity was assessed using variance inflation factors (VIFs) computed on the dummy-coded predictor matrix; all VIFs were <4 (range: 1.04–3.77; maximum VIF = 3.77), indicating no problematic collinearity [19,20].

To evaluate whether the temporal change persisted after adjustment, we additionally modeled calendar period as a three-level indicator (2015–2019 pre-pandemic, 2020–2021 pandemic disruption, 2022–2024 post-2022 recovery) in the fully adjusted modified Poisson model. Period-adjusted prevalence ratios are reported in Supplementary Table S3.

Trend analysis (Joinpoint). To quantify secular changes over 2015–2024, we modeled the annual survey-weighted prevalence using Joinpoint regression for repeated cross-sections. We fitted log-linear models separately for IPRESS use and non-use and evaluated models with 0–2 joinpoints using permutation tests to identify statistically supported changes in slope. We report the Annual Percent Change (APC) for each segment and the Average Annual Percent Change (AAPC) for the overall period with 95% confidence intervals. This approach summarizes population-level changes across calendar years rather than within-person change. Joinpoint models were fitted using the Joinpoint Regression Program (National Cancer Institute) [21]. Two-sided *p* < 0.05 was considered statistically significant. Variance inflation factors (VIFs) for covariates included in the adjusted model (Supplementary Table S2).

As descriptive stratification, we also computed year-specific survey-weighted prevalence of non-use overall and by natural region, and summarized non-use by three periods (2015–2019 pre-pandemic, 2020–2021 pandemic disruption, and 2022–2024 post-2022). These estimates are reported in Supplementary Tables S4 (annual series) and S5 (period-by-region summary).

### 2.6. Ethical Considerations

This research analyzed publicly available, de-identified ENAHO microdata. ENAHO participation is voluntary, and informed consent is obtained by INEI at the time of the interview. The study followed the ethical principles of the Declaration of Helsinki and complied with applicable national regulations governing secondary use of public survey data. The protocol was reviewed and approved by the Institutional Committee for Ethics and Scientific Integrity of Universidad Privada Norbert Wiener (CIEIC-UPNW; Exp. No. A0079-2025; approval date: 12 September 2025). No contact with human participants occurred, and no personally identifiable information was accessed.

### 2.7. Data Availability and Use of Generative AI

ENAHO microdata, questionnaires, and technical documentation are publicly accessible through the INEI microdata portal; no special permissions are required. We analyzed de-identified records only and did not create new data. No generative artificial intelligence tools were used in the design, data collection, statistical analysis, or interpretation of this study. All analytic code followed the prespecified definitions and exclusions described in this section and was applied uniformly to the 2015–2024 files to produce the estimates shown in Table 1, Table 2 and Table 3 and Figure 1.

## 3. Results

Table 1 presents the sociodemographic, clinical, and geographic profile of the 330,165 participants included in the analysis. Women represented 58.9% of the sample, and adults aged 40 to 59 years comprised the largest age group (36.3%). Nearly 45% of respondents had completed only primary education, and 34.7% were married. Regarding health-insurance coverage, 57.0% were insured by SIS, while 17.4% remained uninsured. Two-thirds of participants resided in urban areas, and the highest proportion came from the Andean highlands (42.2%). A total of 65.3% reported a chronic illness, and 9.6% reported a disability. Overall, 58.5% of adults reported using formal health services, whereas 41.5% did not, revealing substantial inequities across demographic and territorial strata.

Figure 1 displays the weighted national trend in IPRESS use and non-use among adults reporting a recent health problem. Non-use was relatively stable before the pandemic (2015–2019), increased sharply during the COVID-19 disruption (2020–2021), and then declined after 2022 but remained above pre-pandemic levels, indicating persistent barriers to effective access and continuity of care (Figure 1). Joinpoint regression identified no statistically supported joinpoints over 2015–2024; accordingly, the AAPC equaled the single-segment APC, corresponding to an average annual increase of +1.07% for non-use and an average annual decrease of −1.51% for IPRESS use (Figure 1).

In covariate-adjusted models, the temporal increase in non-use remained statistically significant. Compared with the pre-pandemic period (2015–2019), non-use was higher during the pandemic disruption (2020–2021; adjusted PR = 1.21, 95% CI: 1.20–1.23) and remained elevated in the post-2022 period (2022–2024; adjusted PR = 1.12, 95% CI: 1.10–1.13), indicating that the observed shift was not explained solely by differences in sex, age, education, insurance, morbidity, disability, or geography (Supplementary Table S3).

Year-specific estimates stratified by natural region are shown in Supplementary Table S4. Non-use increased sharply across all regions during 2020–2021, with the highest levels consistently observed in the Highlands (Sierra). For example, overall non-use rose from 54.7% in 2019 to 68.8% in 2020 and remained high in 2021 (65.9%). Regionally, the pandemic peak reached 68.8% in the Coast (Costa, 2020), 64.3% in Lima Metropolitana (2020), 66.6% in the Amazon (Selva, 2020), and 72.7% in the Highlands (Sierra, 2021). After 2022, non-use declined but remained above pre-pandemic levels across regions (e.g., overall 59.9% in 2024 vs. 54.7% in 2019), indicating incomplete recovery in effective access (Supplementary Table S5).

The bivariate analysis shows significant associations (*p* < 0.001) between non-use of formal health services and most explanatory variables. Percentages in Table 2 are presented as within-group (column) distributions for “Used” and “Did Not Use”. Compared with users, the non-use group included a higher proportion of men (43.3% vs. 37.8%) and younger adults (18–29 years: 18.1% vs. 15.3%). Marked differences were observed by insurance coverage: uninsured adults accounted for 22.9% of non-users versus 9.7% of users, whereas EsSalud coverage was more common among users (30.1% vs. 19.1%) (Table 2).

In the multivariable Poisson regression model, after adjusting for covariates, the likelihood of non-use remained significantly associated with several determinants. Female sex was associated with a lower probability of non-use (PR = 0.92; 95% CI 0.91–0.93). Higher education levels exhibited a clear protective gradient: individuals with tertiary education were 12.5% less likely to forgo care than those without formal schooling. Compared with uninsured participants, those covered by EsSalud and SIS were 35% and 25% less likely, respectively, to omit formal care. Residents of Metropolitan Lima also had a reduced prevalence of non-use (PR = 0.88; 95% CI 0.87–0.90), whereas those living in the highlands showed the highest adjusted prevalence (PR = 1.07; 95% CI 1.06–1.09).

Overall, these adjusted patterns suggest that the pandemic period coincided with a structural upward shift in non-use that persisted beyond 2022, with the highest burden concentrating in the Highlands, highlighting a sustained gap in effective access rather than a purely compositional change in population characteristics.

## 4. Discussion

This study indicates that non-use of formal health services (IPRESS) in Peru remained substantial throughout 2015–2024 among adults reporting recent morbidity, with a pronounced peak during the COVID-19 disruption and an incomplete recovery thereafter. These dynamics are plausible in segmented health systems where entitlement and provider networks are not fully interchangeable and where administrative frictions, waiting times, and territorial supply constraints can prevent timely facility contact despite nominal coverage [16,21]. Consistent with Peru-specific evidence on pandemic-related shocks in access and continuity of care, our findings suggest that persistent inequities by sex, education, insurance, and territory continue to shape effective access, reinforcing the need to move from nominal entitlement to equity-oriented service responsiveness in fragmented systems [9,10].

The observed pandemic-era surge in non-use and only partial reversion thereafter aligns with national analyses showing that unmet need rose sharply during the COVID-19 period and remained elevated beyond the strictest lockdown phase [21]. Service-level evidence also supports the interpretation of uneven recovery: utilization of maternal and child health services dropped abruptly in 2020 and recovered only partially, with stronger disruptions in certain macro-regions [22]. Together, these findings suggest that system shocks can amplify pre-existing territorial and socioeconomic barriers, underscoring the importance of resilience strategies that protect routine care and recovery for populations at highest risk of being left behind in Peru and comparable LMIC settings [9].

Equity gradients were particularly salient for insurance and education. Insurance coverage was consistently protective against non-use, yet the persistence of non-use among insured groups indicates that financial protection alone does not guarantee timely utilization when supply constraints, administrative frictions, waiting times, and indirect costs remain substantial [9]. Similarly, the educational gradient suggests that health literacy, care navigation capacity, and perceived need shape service-seeking behavior. These results converge with evidence of socioeconomic inequalities in healthcare utilization in Peru, where medical consultation use has been shown to concentrate among better-off groups [11]. From a public health perspective, reducing non-use will likely require integrated approaches that combine financial protection with strengthened primary care availability, simplified pathways, and targeted navigation support, with relevance for other countries facing similar inequities [9,11].

Geographic disparities remained prominent. Higher non-use outside Metropolitan Lima plausibly reflects a combination of service availability gaps, travel time and opportunity costs, and uneven distribution of human resources and facility capacity across macro-regions. Importantly, contrasts that change direction after adjustment (e.g., rural vs. urban) should be interpreted as evidence that “place” is intertwined with insurance mix, education, and regional supply rather than as a purely geographic effect. This reinforces that policy responses must be territorially tailored, prioritizing continuity, availability, and quality of first-contact care in high-constraint regions to advance equitable effective coverage in Peru and similar Andean–Amazonian contexts [9].

This study contributes a decade-long, nationally representative assessment of non-use using a harmonized operational definition across 2015–2024, spanning the pre-pandemic baseline, the COVID-19 shock, and the post-2022 recovery. Key strengths include the large sample size and the ability to examine equity-relevant determinants in a consistent framework. Limitations should be considered. First, ENAHO is a repeated cross-sectional survey; therefore, our estimates describe population-level trends and associations but do not support within-person causal inference. Second, morbidity and care-seeking are self-reported over a four-week recall period and may be affected by recall error and differential reporting by education, sex, and region. Third, our outcome captures non-use of facility-based formal services (IPRESS contact) rather than the full construct of unmet need; “care at home” cannot be disaggregated into self-care versus provider home visits, and pharmacy/drugstore contact although regulated occurs outside IPRESS facilities and may include advice and medication without clinical evaluation. Fourth, we lacked direct measures of supply-side constraints (waiting times, staffing, facility readiness, medicine availability) and quality of care, as well as contextual shocks at the local level; residual confounding and mediation by unmeasured factors are therefore possible. Fifth, although we harmonized variables across 2015–2024, changes in survey implementation or in access pathways (e.g., telehealth expansion) may have influenced reporting and comparability over time. Finally, contrasts that attenuate or reverse after adjustment (e.g., rural vs. urban) should be interpreted cautiously, as they may reflect complex collinearity between territory, insurance scheme mix, and socioeconomic position rather than a direct “place effect” [9,10].

## 5. Conclusions

We estimated national trends (2015–2024) and identified factors associated with the non-use of formal health services among Peruvian adults. Non-use remained high overall, increased sharply during the COVID-19 period, and recovered only partially thereafter. After adjustment, non-use was lower among those with health insurance and higher educational attainment, lower in Metropolitan Lima and higher in the Highlands, and modestly lower in rural areas compared with urban areas. Women and older adults were less likely to forgo care, while having a chronic condition was also protective; disability showed no independent association, and partnered adults had lower non-use than singles.

These findings indicate that moving from nominal to effective coverage is critical, particularly through improvements in provider availability, administrative simplicity, and user navigation. Territorial investments in primary care, transport, and referral capacity are needed to close gaps in the Highlands and rainforest settings, while demand-side strategies targeting working-age adults and men can reduce time and access costs. Continuous monitoring of post-pandemic recovery, together with routine equity-sensitive indicators, will be essential to prevent persistent non-use from widening regional and socioeconomic disparities in Peru and comparable middle-income health systems.

## Figures and Tables

**Table 1:** Characteristics of the study population in Peru, 2015–2024.

General Characteristics	N (=330,165)	%
**Sex**		
Male	135,547	41.05%
Female	194,618	58.95%
**Age group**		
18–29 years	55,964	16.95%
30–39 years	52,157	15.80%
40–59 years	119,932	36.32%
≥60 years	102,112	30.93%
**Educational level** ±		
No formal education	31,828	9.65%
Primary	147,578	44.74%
Secondary	84,467	25.61%
Higher	66,002	20.01%
**Marital status**		
Single	56,021	16.97%
Cohabiting	88,254	26.73%
Married	114,409	34.65%
Widowed	31,391	9.51%
Divorced/Separated	40,090	12.14%
**Health insurance**		
Uninsured	57,526	17.42%
EsSalud	78,118	23.66%
SIS	188,211	57.01%
Armed Forces/Police	3436	1.04%
Private insurance	2874	0.87%
**Chronic illness or discomfort**		
No	114,644	34.72%
Yes	215,521	65.28%
**Disability**		
No	298,454	90.40%
Yes	31,711	9.60%
**Area of residence**		
Urban	220,096	66.66%
Rural	110,069	33.34%
**Natural region**		
Coast	94,457	28.61%
Highlands	139,212	42.16%
Rainforest	62,569	18.95%
Metropolitan Lima	33,927	10.28%
**Non-Use of Formal Health Services**		
Did not use	193,225	41.48%
Used	136,940	58.52%

Note: ± Variables with missing data. Social Health Insurance (EsSalud), Comprehensive Health Insurance (SIS). Source: National Household Survey on Living Conditions and Poverty (ENAHO), 2015–2024.

**Table 2:** Bivariate analysis of factors associated with the non-use of formal health services, 2015–2024.

Associated Factors	Non-Use of Formal Health Services				*p*-Value
	Used		Did Not Use		
	n (=136,940)	%	n (=193,225)	%	
**Sex**					<0.001 *
Male	51,813	37.84%	83,734	43.33%	
Female	85,127	62.16%	109,491	56.67%	
**Age group**					<0.001 *
18–29 years	20,929	15.28%	35,035	18.13%	
30–39 years	20,976	15.32%	31,181	16.14%	
40–59 years	48,350	35.31%	71,582	37.05%	
≥60 years	46,685	34.09%	55,427	28.69%	
**Educational level ^†^**					<0.001 *
No formal education	12,507	9.14%	19,321	10.01%	
Primary	59,824	43.73%	87,754	45.45%	
Secondary	34,168	24.98%	50,299	26.05%	
Higher	30,306	22.15%	35,696	18.49%	
**Marital status**					<0.001 *
Single	20,536	15.00%	35,485	18.36%	
Cohabiting	35,147	25.67%	53,107	27.48%	
Married	50,124	36.60%	64,285	33.27%	
Widowed	14,337	10.47%	17,054	8.83%	
Divorced/Separated	16,796	12.27%	23,294	12.06%	
**Health insurance**					<0.001 *
Uninsured	13,303	9.71%	44,223	22.89%	
EsSalud	41,219	30.10%	36,899	19.10%	
SIS	78,992	57.68%	109,219	56.52%	
Armed Forces/Police	1872	1.37%	1564	0.81%	
Private insurance	1554	1.13%	1320	0.68%	
**Chronic disease**					<0.001 *
No	41,425	30.25%	73,219	37.89%	
Yes	95,515	69.75%	120,006	62.11%	
**Disability**					<0.001 *
No	123,317	90.05%	175,137	90.64	
Yes	13,623	9.95%	18,088	9.36	
**Area of residence**					<0.001 *
Urban	94,030	68.67%	126,066	65.24	
Rural	42,910	31.33%	67,159	34.76	
**Natural region**					<0.001 *
Coast	40,336	29.46%	54,121	28.01%	
Highlands	52,946	38.66%	86,266	44.65%	
Rainforest	26,382	19.27%	36,187	18.73%	
Metropolitan Lima	17,276	12.62%	16,651	8.62%	

Note: * Statistically significant (*p* < 0.05); EsSalud: Social Health Insurance; SIS: Comprehensive Health Insurance; Source: National Household Survey on Living Conditions and Poverty (ENAHO), 2015–2024. ^†^ Percentages are calculated based on participants with complete information for educational level.

**Table 3:** Multivariable analysis of factors associated with the non-use of formal health services, 2015–2024.

Associated Factors	Crude PR (95% CI)	*p*-Value	Adjusted PR (95% CI)	*p*-Value
**Sex**				
Male	Reference		Reference	
Female	0.91 (0.90–0.92)	<0.001	0.92 (0.91–0.93)	<0.001
**Age group**				
18–29 years	Reference		Reference	
30–39 years	0.96 (0.94–0.97)	<0.001	1.00 (0.99–1.02)	0.588
40–59 years	0.95 (0.94–0.97)	<0.001	1.02 (1.00–1.04)	0.015
≥60 years	0.87 (0.86–0.88)	<0.001	0.95 (0.93–0.96)	<0.001
**Educational level**				
No formal education	Reference		Reference	
Primary	0.98 (0.96–0.99)	0.009	0.94 (0.92–0.95)	<0.001
Secondary	0.98 (0.96–0.99)	0.023	0.92 (0.89–0.93)	<0.001
Higher	0.89 (0.88–0.91)	<0.001	0.88 (0.86–0.89)	<0.001
**Marital status**				
Single	Reference		Reference	
Cohabiting	0.95 (0.94–0.96)	<0.001	0.96 (0.94–0.97)	<0.001
Married	0.89 (0.88–0.89)	<0.001	0.94 (0.93–0.96)	<0.001
Widowed	0.86 (0.84–0.87)	<0.001	0.94 (0.92–0.96)	<0.001
Divorced/Separated	0.92 (0.90–0.93)	<0.001	0.97 (0.95–0.99)	0.001
**Health insurance**				
Uninsured	Reference		Reference	
EsSalud	0.61 (0.61–0.62)	<0.001	0.65 (0.65–0.66)	<0.001
SIS	0.76 (0.75–0.76)	<0.001	0.76 (0.75–0.76)	<0.001
Armed Forces/Police	0.59 (0.56–0.62)	<0.001	0.64 (0.61–0.67)	<0.001
Private insurance	0.59 (0.57–0.63)	<0.001	0.63 (0.59–0.67)	<0.001
**Chronic disease**				
No	Reference		Reference	
Yes	0.87 (0.86–0.88)	<0.001	0.92 (0.91–0.93)	<0.001
**Disability**				
No	Reference		Reference	
Yes	0.97 (0.96–0.99)	<0.001	1.01 (0.99–1.03)	0.268
**Area of residence**				
Urban	Reference		Reference	
Rural	1.07 (1.06–1.08)	<0.001	0.98 (0.97–0.99)	<0.001
**Natural region**				
Coast	Reference		Reference	
Highlands	1.08 (1.07–1.09)	<0.001	1.07 (1.06–1.09)	<0.001
Rainforest	1.01 (0.99–1.02)	0.168	0.99 (0.97–1.00)	0.072
Metropolitan Lima	0.86 (0.84–0.87)	<0.001	0.88 (0.87–0.89)	<0.001

Abbreviations: PR, prevalence ratio; CI, confidence interval; Statistically significant (*p* < 0.05).

## Data Availability

Publicly available microdata from the National Household Survey on Living Conditions and Poverty (ENAHO), 2015–2024 were analyzed in this study. The datasets are freely available from the INEI public microdata portal; no registration or special permissions are required. No new data were created beyond these publicly available files.

## Supplementary Materials

The following supporting information can be downloaded at: https://www.mdpi.com/article/10.3390/ijerph23020183/s1, Table S1. ENAHO items used, operational definitions, and harmonization/recoding rules for the study outcome and covariates; Table S2. Variance inflation factors (VIFs) for covariates included in the adjusted model; Table S3. Covariate-adjusted association between survey period (2015–2019, 2020–2021, 2022–2024) and non-use of IPRESS (modified Poisson regression; adjusted PR and 95% CI); Table S4. Year-specific survey-weighted prevalence of non-use of IPRESS overall and by natural region (2015–2024); Table S5. Survey-weighted prevalence of non-use of IPRESS by period (2015–2019, 2020–2021, 2022–2024) and natural region.

## Abbreviations

The following abbreviations are used in this manuscript:

ENAHO	National Household Survey (Encuesta Nacional de Hogares, Peru)
INEI	National Institute of Statistics and Informatics (Instituto Nacional de Estadística e Informática, Peru)
IPRESS	Health Service Provider Institution (Institución Prestadora de Servicios de Salud)
MINSA	Ministry of Health (Ministerio de Salud, Peru)
SIS	Comprehensive Health Insurance (Seguro Integral de Salud)
EsSalud	Social Health Insurance of Peru (Seguro Social de Salud)
FFAA/PNP	Armed Forces/National Police Health Services
RP	Prevalence Ratio
CI	Confidence Interval
PSU	Primary Sampling Unit
aPR	Adjusted Prevalence Ratio
IC95%	95% Confidence Interval (Intervalo de Confianza al 95%)
SDOH	Social Determinants of Health
VIF	Variance Inflation Factor
