# Supplementary material for: Trends and Factors Associated with the Non-Use of Formal Health Services in Peru, 2015–2024
Source: Int J Environ Res Public Health. 2026 Jan 31;23(2):183. doi: 10.3390/ijerph23020183 (PMC12940747; doi:10.3390/ijerph23020183)
Supplement: Supplementary file 1 [file ijerph-23-00183-s001.zip › ijerph-4019142-supplementary.pdf]

Table S1. ENAHO items used, operational definitions, and harmonization/recoding rules for the study outcome and covariates.

Variable (manuscript)	Operational definition / recoding used	Original ENAHO recode name(s)
**Outcome: Non-use of formal health services (nusfps)**	Among individuals aged ≥18 who reported any health problem in the last four weeks (p4021–p4024). **Formal use (nusfps = 0):** any of p4031–p4039 = 1 (public or private IPRESS: MINSA, EsSalud, FFAA/PNP, or private clinic). **Non-formal use (nusfps = 1):** none of the above equal 1 and at least one of p40310–p40313 = 1 (pharmacy, home care, no care, or other non-IPRESS option). **Exclusions:** respondents visiting both pharmacy and IPRESS; reporting "not serious/no need" (p4095 = 1); or reporting IPRESS but without specifying provider (p4041–p4047 all missing/zero).	p4021–p4024, p4031–p40313, p4041–p4047, p4095
**Sex**	Male/Female as recorded.	p207
**Age group (years)**	Derived from age (p208a): 18–29, 30–39, 40–59, ≥60.	p208a
**Marital status**	Recoded from p209: 1 = Soltero; 2 = Conviviente; 3 = Casado; 4 = Viudo; 5 = Divorciado/Separado.	p209
**Educational level**	Recoded from p301a: 1 = No education; 2 = Primary; 3 = Secondary; 4 = Higher.	p301a
**Mother tongue / Language**	1 = Spanish; 2 = Quechua, Aymara, or other indigenous.	p300a
**Health insurance**	1 = EsSalud (p4191=1); 2 = SIS (p4195=1); 3 = FFAA/PNP (p4194=1); 4 = Private/other (p4192, p4193, p4196–p4198=1); 0 = None.	p4191–p4198
**Chronic illness**	1 = Yes (p401=1); 0 = No (p401=2).	p401
**Disability**	1 = Any of p401h1–p401h6 = 1; 0 = all = 2.	p401h1–p401h6
**Area of residence**	1 = Urban (estrato in 1–6); 2 = Rural (estrato in 7–8).	estrato
**Natural region**	1 = Costa (dominio 1–3); 2 = Sierra (4–6); 3 = Selva (7); 4 = Lima Metropolitana (8).	dominio
**Household poverty status**	Household categorized as poor/non-poor using INEI classification.	pobreza
**Household wealth quintile**	Derived from socio-economic stratum variable (estrsocial → quintil_riq, 1–5).	estrsocial
**Survey weights**	Individual expansion factor applied for national estimates.	facpob07

Table S2. Variance inflation factors (VIFs) for covariates included in the adjusted model.

Covariate term	VIF
Sex: Female (ref Male)	1.04
Age: 30–39 (ref 18–29)	1.29
Age: 40–59 (ref 18–29)	1.68
Age: ≥60 (ref 18–29)	1.63
Education: Primary (ref No education)	2.77
Education: Secondary (ref No education)	3.55
Education: Higher (ref No education)	3.67
Marital status: Cohabiting (ref Single)	1.76
Marital status: Married (ref Single)	2.07
Marital status: Widowed (ref Single)	1.59
Marital status: Divorced/Separated (ref Single)	1.61
Health insurance: EsSalud (ref Uninsured)	1.91
Health insurance: SIS (ref Uninsured)	2.47
Health insurance: Armed Forces/Police (ref Uninsured)	1.18
Health insurance: Private (ref Uninsured)	1.10
Chronic condition: Yes (ref No)	1.13
Disability: Yes (ref No)	1.11

Area: Rural (ref Urban)	1.28
Natural region: Highlands (ref Coast)	1.38
Natural region: Rainforest (ref Coast)	1.17
Natural region: Metropolitan Lima (ref Coast)	1.15

Table S3. Covariate-adjusted association between survey period (2015–2019, 2020–2021, 2022–2024) and non-use of IPRESS (modified Poisson regression; adjusted PR and 95% CI).

Period	Adjusted PR	95% CI	*p*-value
2015–2019	1.00	Reference	—
2020–2021	1.21	1.20–1.23	<0.001
2022–2024	1.12	1.10–1.13	<0.001

Adjusted for sex, age group, educational level, marital status, mother tongue, health insurance, poverty status, household wealth quintile, chronic illness, disability, area of residence, and natural region. Modified Poisson regression with log link and robust standard errors.

Table S4. Year-specific survey-weighted prevalence of non-use of IPRESS overall and by natural region (2015–2024).

Year	Overall non-use (%)	Costa (%)	Sierra (%)	Selva (%)	Lima Metropolitana (%)
2015	53.7	54.3	61.9	56.4	41.4
2016	56.2	58.1	61.6	56.7	47.4
2017	56.8	57.3	61.9	59.0	49.3
2018	55.6	56.4	60.5	58.3	48.2
2019	54.7	54.8	60.5	56.3	46.8
2020	68.8	68.8	72.6	66.6	64.3
2021	65.9	63.6	72.7	64.2	58.2
2022	61.8	60.0	67.7	60.9	56.7
2023	58.7	56.8	65.6	57.2	55.4
2024	59.9	57.5	64.8	57.3	56.2

Table S5. Survey-weighted prevalence of non-use of IPRESS by period (2015–2019, 2020–2021, 2022–2024) and natural region.

Region	2015–2019 non-use (%)	2020–2021 non-use (%)	2022–2024 non-use (%)
Overall	55.4	67.3	60.1
Coast	56.2	66.2	57.3
Lima Metropolitana	46.7	61.3	54.3
Rainforest	57.3	65.4	59.3
Highlands	61.3	72.7	65.9
